# Sub-chronic exposure to fipronil induced oxidative stress, biochemical and histopathological changes in the liver and kidney of male albino rats

**DOI:** 10.1016/j.toxrep.2015.02.009

**Published:** 2015-02-19

**Authors:** Abdel-Tawab H. Mossa, Eman S. Swelam, Samia M.M. Mohafrash

**Affiliations:** a Environmental Toxicology Research Unit (ETRU), Pesticide Chemistry Department, National Research Centre, 33 Bohouth Street, Dokki, Giza, Egypt; b Economic Entomology and Pesticides Department, Faculty of Agriculture, Cairo University, Egypt

**Keywords:** Fipronil, Rats, Liver, Kidney, Oxidative stress, Histopathology

## Abstract

Fipronil (FPN) is a broad-spectrum *N*-phenylpyrazole insecticide and has been used in agriculture and public health since the mid-1990s. The present study was designed to investigate the adverse effects of sub-chronic exposure to the FPN on the liver and kidney of male rats at three concentrations 0.1, 1 and 10 mg/L in drinking water for 45 days. Serum aspartate aminotransferases (AST), alanine aminotransferases (ALT), alkaline phosphatase (ALP), and lactate dehydrogenase (LDH) activity and levels of uric acid, creatinine and total protein were significantly increased in FPN-treated rats. Oxidative stress biomarkers such as superoxide dismutase (SOD), catalase (CAT), glutathione peroxidase (GPx), glutathione-S-transferase (GST) and glutathione reduced (GSH) were significantly decreased, while lipid peroxidation (LPO) was significantly increased in treating rats in a concentration dependent manner. FPN caused histopathological alterations in liver and kidney of male rats. From our results, it can be concluded that FPN induced lipid peroxidation, oxidative stress, liver, and kidney injury in rats. These pathophysiological changes in liver and kidney tissues could be due to the toxic effect of FPN that associated with a generation of free radicals.

## Graphical abstract

**Figure:**
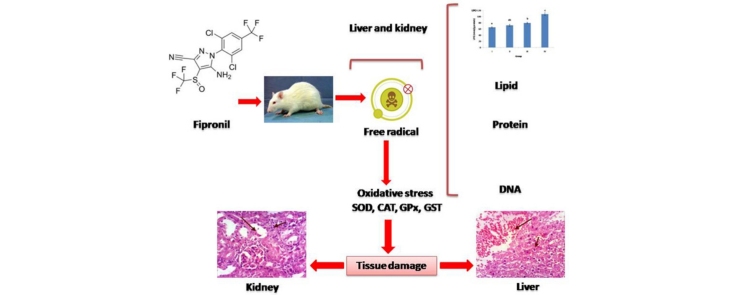

## 1 Introduction

Fipronil (FPN, 5-amino-1-(2,6-dichloro-4-(trifluoromethylphenyl)-4-(trifluoro-methylsulfinyl) pyrazole-3-carbonitrile) is a phenylpyrazole insecticide that is extensively used to control insects in different cereal crops and in public health management [1]. It is more effective than organophosphate, carbamate and pyrethroids insecticides against several species of Lepidoptera, Orthoptera and Coleopteran [2], [3]. Currently, exposure to phenylpyrazole pesticides is a global public health issue and concerns are increased regarding the relative safety of these pesticide groups because of widespread use, their toxicity, and releases into the environment.

FPN is neurotoxic to insects and the primary mechanism of action refers to blocks ion tropic gamma-amino butyric acid receptor (GABAR) of the central nervous system that causes hyper-excitation at low doses and convulsions leading to insect death at high doses [4]. FPN is more toxic to insects than mammals [5] and has moderately acute oral toxicity LD_50_'s ranging from 40 to 100 mg/kg body weight in rats and mice [3], [6]. Therefore, complete selectivity of pesticides is difficult and most of the pesticides are toxic to non-target organisms, including humans [7].

FPN is a strong uncoupler of oxidative phosphorylation at relatively low concentrations in SH-SY5Y human neuroblastoma cells in vitro and induced neuronal apoptosis, mediated by increased generation of reactive oxygen species (ROS) [8]. Both HepG2 cells and primary human hepatocytes are sensitive to the cytotoxic effects of FPN [9]. FPN and its metabolites induced cytotoxicity in epithelial model Caco-2 cells at micromolar concentration exposure [8]. FPN inhibits DNA and protein synthesis in rat neuronotypic pheochromocytoma PC12 cells and induced oxidative stress more than chlorpyrifos [10]. FPN causes endocrine disruption and adverse reproductive effects in female rats [11], elevation in lipid peroxidation (LPO) and decrease in the levels of glutathione (GSH) at the dosage of 0.5 mg/kg/day for 98 days to buffalo calves [12] and altered SOD and CAT activities in the liver of *Cyprinus carpio* [13]. It decreased total thyroxine (T4), increased hepatic enzymes in plasma of female rat [14], and caused acute human poisoning [15], [16].

Liver and kidney are the most sensitive and main target organs of pesticide toxicity and damage [17], they play a major role in the biotransformation of pesticides. The sensitivity of these tissues to this stress to pesticides is a function of the disturbed balance between the degree of oxidative stress and the antioxidant capability [17], [18]. Previous studies show that pesticides alter enzymatic and non-enzymatic antioxidant and induced oxidative stress in animals that was investigated as a potential mechanism of pesticide toxicity [16], [18], [19]. It has been reported that prolonged exposure to low doses of fipronil leads to oxidative stress in serum of pregnant rats and their offspring [20].

Pesticide formulations are complex mixtures that contain, besides the active ingredient(s), several other components, such as solvent, wetting, emulsifying agents, and additives; therefore the toxicity information on active ingredients alone is not sufficient to evaluate the adverse health effects of commercial pesticides. Therefore, the WHO emphasized the necessity of evaluating toxic hazard of the formulated pesticides [21]. Over the last decade, the usage of FPN has increased considerably and information on adverse health effects is very limited. To the best of our knowledge, there are no published studies that have examined the effect of formulated FPN on oxidant/antioxidant status and the liver and kidney function biomarkers in male rats. Therefore, this study aimed to evaluate the adverse effects of sub-chronic exposure to formulated FPN on oxidant/antioxidant status and liver and kidney biomarkers of male rats.

## 2 Materials and methods

### 2.1 Animals and management

Male albino rats weighing 105 ± 5 g were procured from the Animal Breeding House of the National Research Centre (NRC), Dokki, Giza, Egypt. Rats were housed in polypropylene cages (six rats in each), with free access standard pellet diet, water ad libitum, under standardized housing conditions (12 h light/dark cycle, temperature (23 ± 2 °C) and a minimum relative humidity of 48% in the laboratory. The rats were acclimatized for 1 week before the start of the experiment. All the rats were kept according to the guidelines and welfare regarding animal protection approved by NRC Local Ethical Review Committee and was conducted in accordance with the “Guide for the Care and Use of Laboratory Animals”.

### 2.2 Chemicals and reagents

Fipronil (Insecto SC 5%) is a product of BASF Company and manufactured by, Sinochem Group Ningbo Technical Co., Ltd., China. The assay kits used for biochemical measurements of aspartate aminotransferases (AST, EC 2.6.1.1.), alanine aminotransferases (ALT, EC 2.6.1.2), alkaline phosphatase (ALP, EC 3.1.3.1), lactate dehydrogenase (LDH, EC 1.1.1.27), superoxide dismutase (SOD, EC 1.15.1.1), catalase (CAT, EC 1.11.1.6), glutathione peroxidase (GPx, EC 1.11.1.9), glutathione-s-transferase (GST, EC 2.5.1.13), glutathione reduced (GSH), lipid peroxidation (LPO), albumin, uric acid and creatinine were purchased from Biodiagnostic Company, Dokki, Giza, Egypt. All other chemicals were of reagent grades and were obtained from reputed companies.

### 2.3 Experimental design

Rats were randomly divided into four experimental groups, six rats. Group I, received water and served as a control. The remaining three groups (II, III and IV) received FPN in drinking water at concentrations 0.1, 1.0 and 10 mg/L for 45 consecutive days. The concentrations of FPN were calculated depending on the percentage of active ingredients of commercial formulation of FPN. Concentrations of FPN were freshly prepared and body weights were monitored weekly during the experimental period. All rats were observed for signs of toxicity and mortality daily for 45 days.

The concentrations used in this study represent 2.0, 0.2 and 0.02 mg/kg b.wt. of FPN, based on average water consumptions and body weights of treated rats. The lower concentration of FPN represents the dose of no observable adverse effect level (NOAEL) of human [22] with descending concentration levels by 10-fold interval, i.e., 1 and 10 mg/L.

### 2.4 Blood and tissue samples

At the end of the experimental period, rats were fasted overnight and blood samples were collected by puncturing the retero-orbital venous plexus of the animals with a fine sterilized glass capillary, then rats were sacrificed by cervical dislocation. Blood samples were left to clot in clean dry tubes and centrifuged at 3000 rpm (600 x *g*) for 10 min at 4 °C using Heraeus Labofuge 400R (Kendro Laboratory Products GmbH, Germany) to obtain the sera. Serum samples were stored at −20 °C for further biochemical analysis, such as AST, ALT, ALP and LDH.

Liver and kidney were excised immediately after sacrificed, cleaned in saline and weighed. Small pieces of each liver and kidney were cut and kept in 10% natural formalin for histopathological study. The other portions of liver and kidney were homogenized in 10% (w/v) ice cold 100 mM phosphate buffer (pH 7.4) and centrifuged at 10,000 rpm (2000 × *g*) for 15 min at 4 °C, and then the supernatant was obtained and used for oxidative stress biomarkers studies (SOD, CAT, GPx, GSH, LPO) and total protein.

### 2.5 Serum biochemical parameters

#### 2.5.1 Liver and kidney function biomarkers

Serum AST and ALT were determined according to the methods of Reitman and Frankel [23], ALP according to Young et al. [24], LDH as an indicator of necrotic cell death was determined according to Vassault [25], albumin, uric acid and creatinine according to Westgard and Poquette [26], Tietz et al. [27] and Tietz [28], respectively. All serum biomarkers were performed according to the details given in Biodiagnostic kit's instructions (Biodiagnostic Company, Dokki, Giza, Egypt).

#### 2.5.2 Oxidative stress biomarkers in liver and kidney

Determination of SOD, CAT, GPx, GST, GSH and lipid peroxidation (LPO) were performed according to the details given in Biodiagnostic kit's instructions and the principals below of different methods are given for each concerned biochemical parameter.

SOD was determined according to the method of Nishikimi et al. [29]. The method based on the ability of SOD enzyme to inhibit the phenazine methosulphate-mediated reduction of nitroblue tetrazolium dye. Briefly, 0.05 ml sample was mixed with 1.0 ml buffer (pH 8.5), 0.1 ml nitroblue tetrazolium (NBT) and 0.1 ml NADH. The reaction was initiated by adding 0.01 ml phenazine methosulphate (PMs), and then absorbance was read at 560 nm for 5 min. SOD activity was expressed as units/mg protein.

CAT was determined according to the method of Abei [30]. The method is based on the decomposition of H_2_O_2_ by catalase. The sample containing catalase is incubated in the presence of a known concentration of H_2_O_2_. After incubation for exactly 1 min, the reaction is quenched with sodium azide. The amount of H_2_O_2_ remaining in the reaction mixture is then determined by the oxidative coupling reaction of 4-aminophenazone (4-aminoantipyrene, AAP) and 3,5-dichloro-2-hydroxybenzenesulfonic acid (DHBS) in the presence of H_2_O_2_ and catalyzed by horseradish peroxidase (HRP). The resulting quinoneimine dye (N-(4-antipyrl)-3-chloro-5-sulfonate-p-benzoquinonemonoimine) is measured at 510 nm. CAT activity was expressed as μmol/mg protein.

GST was determined according to the manufacturer's instructions referred to Habig et al. [31]. The method was based on the conjugation of 1-chloro-2,4-dinitrobenzene (CDNB) with reduced glutathione (GSH) in a reaction catalyzed by GST. GST activity was expressed as μmol/mg protein.

GPx was determined spectrophotometrically according to the method of Paglia and Valentine [32]. The estimation of GPx activity was based on the oxidation of GSH and NADPH using glutathione reductase (GR) and measuring the decrease in absorbance at 340 nm and expressed in units/mg protein.

GSH level was assessed spectrophotometrically according to the method of Beutler et al. [33]. The method was based on the reduction of 5,5′-dithiobis(2-nitrobenzoic acid) (DTNB) with glutathione to produce a yellow compound. The reduced chromogen directly proportional to GSH concentration and its absorbance can be measured at 405 nm. GSH content was expressed in μmol/mg protein.

Lipid peroxidation was estimated by measuring thiobarbituric acid reactive substances (TBARS) and was expressed in terms of malondialdehyde (MDA) content by a colorimetric method according to Satoh [34]. The MDA values were expressed as nmoles of MDA/g protein.

Protein concentration in homogenate was determined according to the method described by Lowry et al. [35].

#### 2.5.3 Histopathological studies

Liver and kidney were removed and dehydrated in graded serial of alcohol and embedded in paraffin wax [36]. Five micrometer thick sections were cut and stained by hematoxylin and eosin (H&E). One slide was prepared for each organ; each slide contained two sections and ten field areas were examined for histopathological changes. The examination was done using a light microscope (Olympus BX50) with a digital camera (Olympus E-410). The histopathological alterations in liver and kidney tissues were scored as follows: normal appearance (−), mild (+), moderate (++) and severe (+++) [36].

#### 2.5.4 Statistical analysis

All data were expressed as mean ± standard error (SE). Data were statistical analyzed by one-way ANOVA analysis followed by Duncan's test using SPSS version 17.0 for windows and the differences were statistically significant at *p* < 0.05.

## 3 Results

### 3.1 Signs of toxicity

No mortality occurred during the experimental period. Generally, signs of toxicity included a change in activity and abnormal gait were observed in rats exposed to concentration 10 mg/L of FPN from the second week.

### 3.2 Body and relative organ weights

**Fig. 1:**
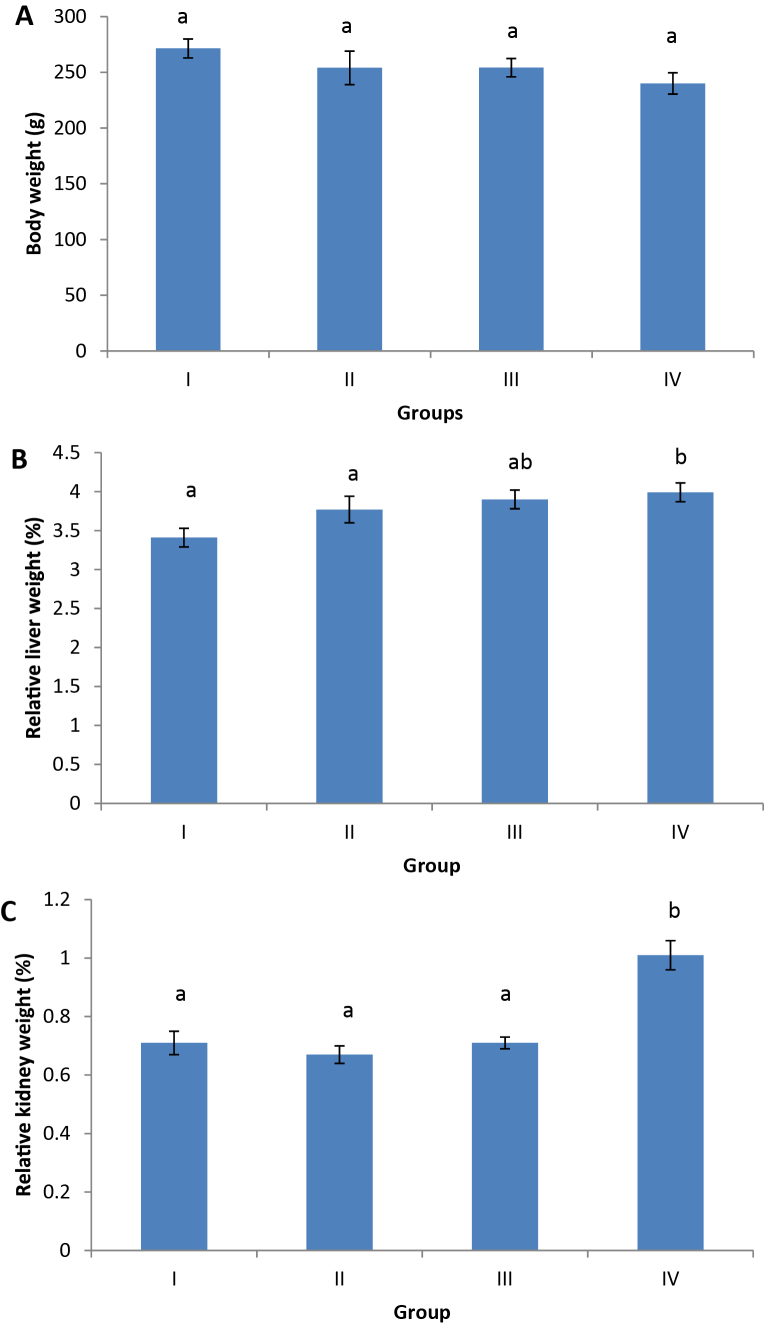
Body weight (A) and relative weights of liver (B) and kidney (C) of male rats exposed 0 1, 1.0 and 10.0 mg/L of fipronil (groups II, III and IV, respectively) for 45 days in drinking water and control group (I). Values represented are means ± SE. Means having the same letters are not significantly different from each other, *p* < 0.05. Relative organ weight = [organ weight/body weight] × 100.

Data of final body weights and relative liver and kidney weights of male rats subjected to different treatments are shown in Fig. 1. A slight decrease in the body weight after 45 days of FPN treatment with the three concentrations, compared to control group. The weekly body weight gain was insignificant changed between treatments. As observed in the case of relative liver and kidney weights (Fig. 1B and C), both organ weights were found to have significant alterations in rat exposed to 10 mg/L of FPN. Moreover, the relative liver and kidney weights of rat exposed to 1 mg/L and 0.1 mg/L were altered slightly.

### 3.3 Serum biochemical parameters

The activity of serum enzymes; AST, ALT, ALP and LDH was significantly increased after 45 days of exposure to 1 and 10 mg/L of FPN when compared to the control group (Table 1). On the other hand, FPN at 0.1 mg/L increased the activity of ALT compared to untreated one, while the activities of AST, ALP and LDH had no significant changes.

**Table 1:** The activity of some enzymes in the sera of male rats exposed to the sub-lethal concentrations of fipronil for 45 days.

Treatments	AST (U/L)	ALT (U/L)	ALP (U/L)	LDH (U/L)
I	52.98 ± 0.76^a^	30.60 ± 0.93^a^	60.24 ± 0.98^a^	112.25 ± 4.12^a^
II	54.76 ± 0.53^a^	34.62 ± 0.76^b^	64.81 ± 1.11^a^	116.95 ± 4.28^a^
III	58.50 ± 0.54^b^	47.40 ± 0.72^c^	70.01 ± 1.74^b^	150.08 ± 4.51^b^
IV	79.20 ± 1.16^c^	63.10 ± 1.23^d^	102.75 ± 2.53^c^	202.70 ± 6.79^c^

Each value is a mean of six animals ± SE. Means having the same letters are not significantly different from each other, *p* < 0.05. I: control group; II, III and IV: groups; animal that received 0.1, 1.0 and 10.0 mg/L of FPN respectively.

As shown in Table 2 the comparative analysis of total protein, uric acid and creatinine in group IV was significantly increased. On the other hand, significant increase in total protein and creatinine were recorded in rats exposed to 1 mg/L of FPN (group III). The results revealed that FPN changed serum biomarkers in a concentration dependent manner.

**Table 2:** Effect of fipronil on albumin, total protein, uric acid and creatinine levels in sera of male rats.

Treatments	Total protein (g/dl)	Albumin (g/dl)	Uric acid (mg/dl)	Creatinine (mg/dl)
I	6.79 ± 0.21^a^	4.79 ± 0.09^a^	6.31 ± 0.28^a^	0.95 ± 0.02^a^
II	7.21 ± 0.19^ab^	4.78 ± 0.06^a^	6.58 ± 0.22^ab^	0.99 ± 0.03^a^
III	7.52 ± 0.16^b^	4.74 ± 0.10^a^	7.02 ± 0.31^ab^	1.18 ± 0.04^b^
IV	8.51 ± 0.32^c^	4.53 ± 0.11^a^	7.25 ± 0.16^b^	1.37 ± 0.03^c^

Each value is a mean of six animals ± SE. Means having the same letters are not significantly different from each other, *p* < 0.05. I: control group; II, III and IV: groups; animal that received 0.1, 1.0 and 10.0 mg/L of FPN respectively.

### 3.4 Oxidative stress biomarkers

**Fig. 2:**
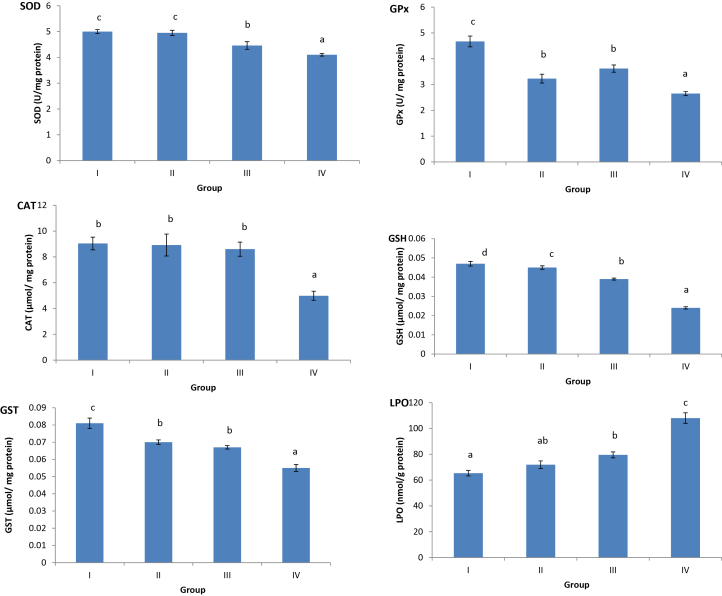
Oxidative stress biomarkers; SOD, CAT, GPx, GST, GSH, LPO in kidney tissue of male rats exposed to 0 1, 1.0 and 10 mg/L of fipronil (groups II, III and IV, respectively) for 45 days in drinking water and control group (I). values represented are means ± SE. Means having the same letters are not significantly different from each other, *p* < 0.05.

As shown in Table 3 and Fig. 2, oxidative stress biomarkers; SOD, CAT, GST, GPx, GSH and LPO were determined in liver and kidney tissues of male rats exposed to 0.1, 1 and 10 mg/L of FPN for 45 days. Rats exposed to 1 mg/L of FPN (group III) showed significant changes in the activity of GPx, GSH and LPO level in liver tissue and SOD, GST, GPx, GSH activities and LPO level in kidney tissue. In group IV, rats exposed to 10 mg/L of FPN, significant changes in all oxidative stress biomarkers (SOD, CAT, GST, GPx, GSH and LPO) were recorded in both organs. The present results revealed that FPN caused statistically significant changes in oxidative stress biomarkers in the liver and kidney homogenates in a concentration dependent manner.

**Table 3:** Effect of fipronil on oxidative stress biomarkers in liver tissues of male rats.

Treatments	Oxidative stress biomarker					
	SOD (U/mg protein)	CAT (μmol/mg protein)	GST (μmol/mg protein)	GPx (U/mg protein)	GSH (μmol/mg protein)	LPO (nmol/g protein)
I	6.67 ± 0.11^b^	13.85 ± 0.25^b^	0.43 ± 0.017^b^	7.78 ± 0.34^c^	0.078 ± 0.002^c^	74.25 ± 2.44^a^
II	6.82 ± 0.14^b^	13.56 ± 0.42^b^	0.42 ± 0.007^b^	5.87 ± 0.31^b^	0.076 ± 0.002^c^	78.17 ± 3.23^ab^
III	6.86 ± 0.23^b^	12.98 ± 0.28^b^	0.41 ± 0.004^b^	5.23 ± 0.39^b^	0.071 ± 0.0013^b^	87.62 ± 2.56^b^
IV	5.47 ± 0.07^a^	7.91 ± 0.26^a^	0.33 ± 0.012^a^	3.52 ± 0.27^a^	0.048 ± 0.002^a^	111.76 ± 4.21^c^

Each value is a mean of six animals ± SE. Means having the same letters are not significantly different from each other, *p* < 0.05. I: control group; II, III and IV: groups; animal that received 0.1, 1.0 and 10.0 mg/L of FPN, respectively.

### 3.5 Histopathological observations

**Fig. 3:**
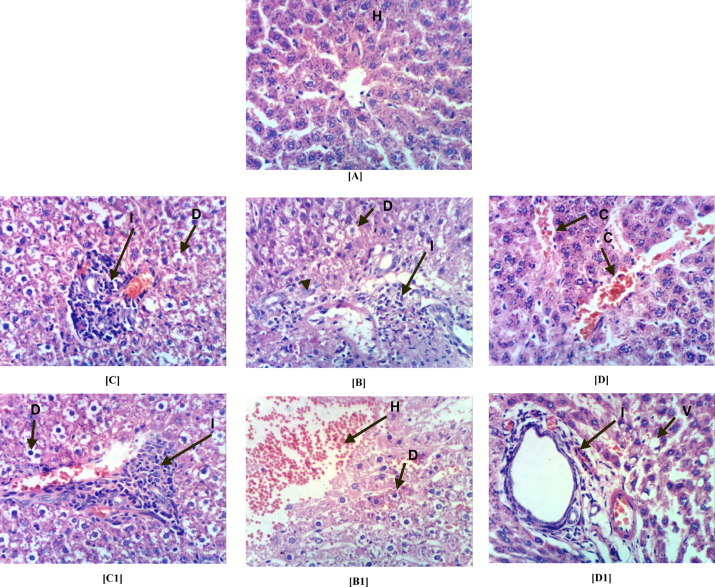
Photomicrograph of liver sections stained by H&E for histopathological changes showing: control (group I) showing (A) the normal histopathological structure of hepatic lobule (H) (64×). 10 mg/L of FPN (group IV) showing (B) ballooning degeneration (D) of hepatocytes, portal infiltration with inflammatory (I) cells and oval cell proliferation (400×) and (B1) showing ballooning degeneration (D) of hepatocytes and focal hepatic hemorrhage (H) (400×). FPN at 1.0 mg/L (group III) showing (C and C1) degeneration of hepatocytes and portal infiltration with inflammatory cells (400×). FPN at 0.1 mg/L (group II) showing (D) congestion (C) of central vein (400×) and (D1) cytoplasmic vacuolization (V) of focal hepatocytes and cystic dilation of bile duct (400×).

**Fig. 4:**
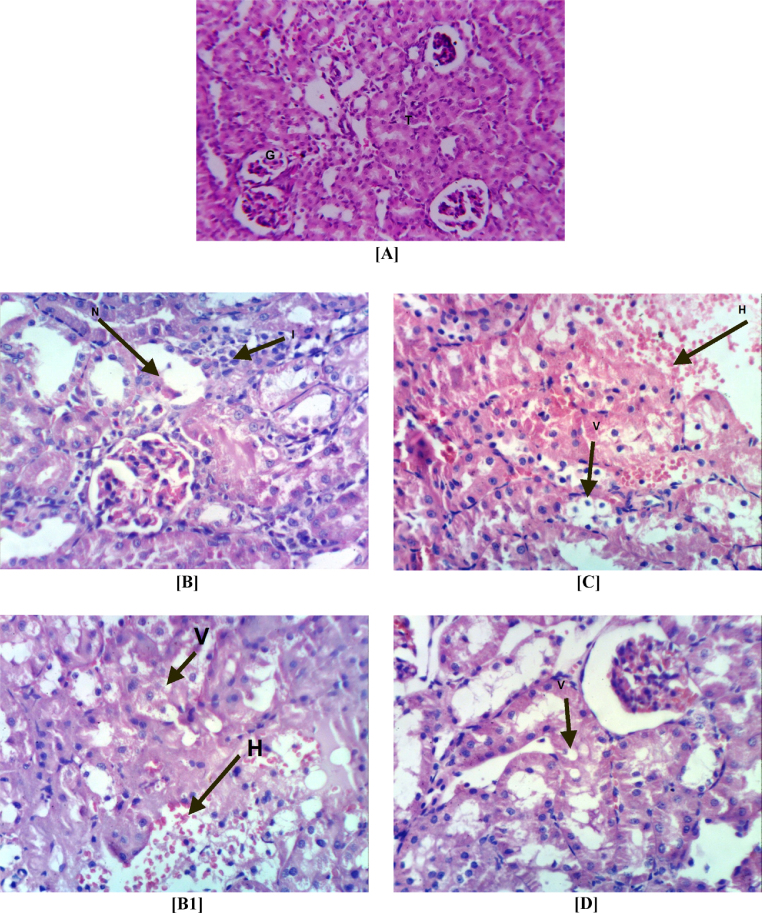
Photomicrograph of kidney sections stained by H&E for histopathological changes showing: control (group I) showing (A) the normal histopathological structure of the glomerular (G) and tubules (T) at the cortex (64×). 10 mg/L of FPN (group IV) showing (B) focal necrosis (N) of renal tubules associated with inflammatory (I) cells infiltration as well as atrophy of glomerular tuft (400×) and (B1) vacuolation (V) of epithelial lining renal tubules and focal hemorrhage (H) (400×). FPN at 1.0 mg/L (group III) showing (C) vacuolation (V) of epithelial lining renal tubules and focal hemorrhage (H) (400×). FPN at 0.1 mg/L (group II) showing (D) vacuolation (V) of epithelial lining renal tubules (400×).

Fig. 3, Fig. 4 and Table 4 show histopathological alterations and score in the liver and kidney of male rats exposed to 0.1, 1 and 10 mg/L of FPN for 45 days compared to control. Liver sections in the control rats (group I) showed the normal histopathological structure of the hepatic lobule (Fig. 3a). Severe histopathological alterations, including degeneration, infiltration, inflammatory cells, cell proliferation and focal hepatic hemorrhage were noted in the liver of male rats exposed to 10 mg/L of FPN (group IV) (Fig. 3b). Rats exposed to 1 mg/L of FPN (group III) showed degeneration of hepatocytes and portal infiltration with inflammatory cells (Fig. 3c). Mild alterations, including, congestion, vacuolization and cystic dilation of bile duct were noted in the liver of male rats exposed to 0.1 mg/L of FPN (group II) (Fig. 3d).

**Table 4:** The severity of the reaction in liver and kidney tissue of different groups according to the histopathological alterations.

Histopathological alterations	Treatments			
	I	II	III	IV
*Liver*				
Ballooning degeneration of hepatocytes	–	+	+++	+++
Congestion of central vein	–	+	–	–
Portal infiltration with inflammatory cells	–	+	++	+++
Oval cells proliferation	–	–	–	++
Focal hepatic hemorrhage	–	–	–	++
Cystic dilatation of bile duct	–	–	–	–

*Kidneys*				
Focal necrosis of renal tubules associated with inflammatory cells infiltration.	–	–	–	+++
Vacuolation of epithelial lining renal tubules.	–	+	++	+++
Focal renal hemorrhage	–	–	++	++

+++ Severe; ++ moderate; +mild; – nil. I: control group; II, III and IV: groups received FPN at concentrations of 0.1, 1.0 and 10.0 mg/L.

Light microscopic of kidney sections in the control rats (group I) showed the normal histopathological structure of renal parenchyma (Fig. 4a). Severe histopathological alterations, including necrosis, inflammatory cell infiltration, atrophy of glomerular tuft, vacuolation, focal hemorrhage (Fig. 4b) were noted in the kidney of male rats exposed to 10 mg/L of FPN (group IV). Rats exposed to 1 mg/L of FPN (group III) showed vacuolation of epithelial lining renal tubules and focal hemorrhage (Fig. 4c). Kidney of rats exposed to 0.1 mg/L of FPN (group II) appeared as normal control with mild vacuolation of epithelial lining renal tubules (Fig. 4d).

## 4 Discussion

No mortality was observed in rats exposed to the FPN at different concentrations for 45 days. While the clinical signs related to FPN-treatment (group IV) included a change in activity and abnormal gait were observed from the second week. The potential for FPN to induce specific neurotoxicity was reported by other studies in mice [37], rats [38] and human [16].

In the present study, exposure of rat to the FPN resulted in a slightly reduced body weight and significant elevation in relative liver and kidney weights especially at high concentration (group IV). The slight decrease in body weight may be due to the oxidative stress and neurotoxicity of FPN, while the increase in relative liver and kidney weights could be attributed to the relationship between the liver weight increase and various toxicological effects or to the reduction in body weight gain of experimental animals [17], [18], [19], [39]. FPN treatment caused an increase in liver and thyroid gland weight of rats [40].

The results of the present study indicate that sub-chronic exposure to 1 and 10 mg/L of FPN (groups III and IV) cause liver and kidney damage in treated rats compared to control as shown by increases in serum marker enzymes AST, ALT, ALP and LDH along with increases in creatinine, uric acid and total protein levels in a concentration dependent manner. Liver enzymes in serum e.g., AST, ALT, ALP and LDH are mainly used in the evaluation of hepatic damage. Transaminases (AST and ALT) play an important role in amino acids catabolism and biosynthesis. They are responsible for detoxification processes, metabolism and biosynthesis of energetic macromolecules for different essential functions [41], [42] and used as specific indicators for liver damage [43]. The increase in these enzymes may be due to liver dysfunction and disturbance in the biosynthesis of these enzymes with alteration in the permeability of the liver membrane takes place [44]. In the current study, the elevation in LDH activity in serum of rat exposed to FPN may be due to the hepatocelluar necrosis and leakage of the enzyme into the blood [45]. Oral administration of FPN at dose 0.5 mg kg^−1^ day^−1^ for 21 days induced significant increases in plasma LDH, AST, acid phosphatase, gamma-glutamyl transferase, total plasma proteins and blood glucose in male buffalo calves [46]. The results are supported by other studies conducted on others insecticides [17], [18], [39], [45], [47], [48].

Serum uric acid and creatinine concentrations were significantly increased in FPN-treated rats (group IV). The increased in uric acid may be due to degradation of purines and pyrimidines or increase uric acid concentration by either overproduction or the inability of excretion [39], [47] and an elevation of creatinine level in the blood is thus an indication of impaired kidney function [49], [50]. In addition, the increase of total protein in FPN-treated rats (groups III and IV) may be due to the liver and kidney dysfunctions, partially because of the high elevation of the serum enzymes [17], [18], [39]. Other investigations showed an increase of urea, creatinine and total protein in the serum in rats exposed to different pesticides [43], [44], [49]. Rat organs affected by chronic FPN exposure include the thyroid, liver and kidney [51].

Results revealed that FPN treatment caused oxidative stress in the liver and kidney of male rats, which is evident from the generation of lipid peroxidation (LPO). LPO is known to disturb the integrity of cellular membranes and implicated in the pathogenesis of various liver and kidney injuries [52], [53]. Therefore, it has used as biomarkers of pesticides induced oxidative stresses [54] and suggested as one of the molecular mechanisms involved in pesticides-induced toxicity [55]. Increased levels of malondialdehyde (MDA), a lipid peroxidation produced in the liver and kidney of FPN-treated rats may be due to increased production of reactive oxygen metabolites, especially hydroxyl radicals and alter antioxidant defense system [19]. Tukhtaev et al. [20] found that prolonged exposure to low doses of FPN increased LPO in liver of pregnant rats and their offspring. Available studies indicate that insecticides increased LPO in animals [45], [52], [53].

FPN-treatment increased oxidative stress by altering the enzyme activities associated with antioxidant defense mechanisms in the liver and kidney of male rats. It caused decreases in the activity of antioxidant enzymes SOD, CAT, GPx and GST and level of GSH in a concentration dependent manner. Enzymatic and non-enzymatic antioxidants work together to prevent the effect of ROS in tissues and are active in the defense against oxidative cell injury by means of their being free radical scavengers [56]. Therefore, SOD, CAT and GPx are considered first defenses that protect cell macromolecules from oxidative damage. In this respect, SOD accelerates the dismutation of superoxide anion to less reactive molecule (H_2_O_2_) which is rapidly converted to water and oxygen by CAT and GPx [52], [57]. In the present study, the decreased in SOD, CAT and GPx activity in the liver and kidney in rats exposed to FPN could be due to excess production of O_2_•^−^ which rapidly converted to H_2_O_2_ by SOD and to water by CAT and GPx. Previous studies reported that pesticides e.g. chlorpyrifos, triazophos leads to decrease in SOD, CAT and GPx in liver and kidney of rats [17], [18], [21], [45], [52], [53], [57]. In agreement with our studies, a significant decrease in SOD activity was observed in liver of pregnant rats and their offspring exposed to low doses of FPN [21], also SOD, CAT and GPx activity were decreased by diazinon treatment in mice [58]. FPN can be responsible for increases in the production of reactive oxygen species (ROS) in cells, which lead to increased lipid peroxidation levels and oxidative stress [63]. It has been reported that FPN was responsible for oxidative stress in *C. carpio*, which was evident through alterations on antioxidant enzymes and increased lipid peroxidation levels [59].

GST constitute a family of enzymes that play an important role in metabolism, particularly in detoxification of xenobiotic e.g. pesticides and intracellular transport of metabolites [60]. These enzymes catalyze the conjugation of reactive electrophiles with glutathione (GSH), and have been implicated with the potential of forming reactive intermediates in particular when GSH levels in the cells are attenuated, consequently resulting in toxicological effects [61], [62]. In the present study, the activity of GST and the level of GSH were significantly decreased in liver and kidney of FPN-treated rats (groups III and IV). The decrease in antioxidant enzyme activities in liver and kidney tissues was due to cellular injury and death of healthy cells that are able to respond to the oxidative insult. In addition, it may be due to the insufficient detoxification capacity of FPN and damage caused by reactive oxygen species. FPN reduced the activity of GST and GPx in tadpoles of frogs [60]. Similar significant decreased in GST activity were observed in the liver of chlorpyrifos treated rats [17].

The change in oxidative stress, liver and kidney biomarkers in rats exposed to the FPN corroborated the histopathological lesions observed in this study. The histological observations of liver reveal severe degeneration, infiltration, inflammation and focal hepatic hemorrhage. In kidney severe necrosis, inflammation, atrophy of glomerular tuft, vacuolation and focal hemorrhage were observed. These observations indicated marked changes in the overall histoarchitecture of liver and kidney in response to FPN. These changes could be due to FPN toxic effects primarily by the generation of reactive oxygen species causing damage to the various membrane components of the cell. Histological observations on the liver and kidney of FPN treated rats were comparable with other studies conducted on chlorpyrifos [18], malathion [63], methyl parathion [64], fenitrothion [65] and other insecticides [66] that reveal insecticides are known to induce a number of histopathological changes in liver and kidney. The observations in the previous mentioned studies are in corroboration and support our results.

## 5 Conclusion

In view of the data of the present study, it can be suggested that FPN induced liver and kidney damage that corroborated with the histopathological lesions. FPN exposure produced marked elevations in LPO and alterations in antioxidant biomarkers in liver and kidney tissues. Therefore, the changes in liver and kidney functions could be due to the generation of ROS, which causing damage to membrane and all cell components.

## Conflict of interest

None declared.

## Transparency document

Transparency document
